# Suppression of NOX2-Derived Reactive Oxygen Species (ROS) Reduces Epithelial-to-MesEnchymal Transition Through Blocking SiO_2_-Regulated JNK Activation

**DOI:** 10.3390/toxics13050365

**Published:** 2025-04-30

**Authors:** Guanhan Xiang, Liang Gong, Kai Wang, Xiaobo Sun, Zhihong Liu, Qian Cai

**Affiliations:** 1 School of Public Health, Ningxia Medical University, No. 1160, Shengli Street, Xingqing District, Yinchuan 750101, China; xgh0903@nxmu.edu.cn (G.X.); m18295128578@163.com (L.G.); wangk0205@163.com (K.W.); sunxiaobo1314520@163.com (X.S.); 2 Key Laboratory of Environmental Factors and Chronic Disease Control, No. 1160, Shengli Street, Xingqing District, Yinchuan 750101, China

**Keywords:** silicosis, epithelial–mesenchymal transition, nicotinamide-adenine dinucleotide phosphate oxidase 2, reactive oxygen species, junk-signal pathway

## Abstract

(1) Background: Silicosis, a chronic lung fibrosis disorder triggered by the accumulation of silica dust in the deep lung regions, is characterized by intricate molecular mechanisms. Among these, the NOX2 (NADPH oxidase 2) and JNK (C-Jun N-terminal kinase) signaling pathways play pivotal roles in the progression of pulmonary fibrosis. Despite their significance, the precise mechanisms underlying the crosstalk between these pathways remain largely unexplored. (2) Methods: To unravel these interactions, we examined the interplay between JNK and NOX2 in human epithelial cells subjected to silica dust exposure through in vivo assays, followed by validation using single-cell sequencing. Our findings consistently revealed elevated expression levels of key components from both the JNK signaling pathway and NOX2 in the lungs of silicosis-induced mice and silica-treated human epithelial cells. (3) Results: Notably, the activation of these pathways was linked to increased ROS (reactive oxygen species) production, elevated levels of profibrogenic factors, and diminished cell proliferation in silica-exposed human lung epithelial cells. Further mechanistic analyses demonstrated that JNK signaling amplifies NOX2 expression and ROS production induced by silica exposure, while treatment with the JNK inhibitor SP600125 mitigates these effects. Conversely, overexpression of NOX2 enhanced silica-induced JNK activation and the expression of epithelial–mesenchymal transition (EMT)-related factors, whereas NOX2 knockdown exerted the opposite effect. These results suggest a positive feedback loop between JNK and NOX2 signaling, which may drive EMT in lung epithelial cells following silica exposure. (4) Conclusions: This reciprocal interaction appears to play a critical role in lung epithelial cell damage and the pathogenesis of silicosis, shedding light on the molecular mechanisms underlying profibrogenic disease and offering potential avenues for therapeutic intervention.

## 1. Introduction

Silicosis is a chronic, irreversible interstitial lung disease that develops due to inhaling free crystalline silica (CS) dust [1]. In work environments such as mining, pottery production, glass manufacturing, and concrete production, millions of workers worldwide face the huge risk of silicosis [2]. Once exposed to air dust for a long time, free silicon dioxide particles enter the respiratory tract and react with multiple cell types. This then triggers a series of adverse consequences, such as cell activation, inflammation, and oxidative stress [3]. Moreover, the metabolites generated will further enhance fibroblast recruitment, promote mesenchymal cell proliferation, and stimulate the release of extracellular matrix components [4]. Subsequently, this will cause progressive pulmonary fibrosis, continuously aggravate lung damage, and ultimately lead to respiratory failure [1,5,6]. Silicosis is one of the most serious and widespread occupational diseases, and the mechanisms of the pathogenic process of silicosis remain unclear [7].

NADPH oxidase (NOX) is an enzyme complex composed of the membrane-bound subunits gp91phox (NOX2) and p22phox, along with the cytoplasmic subunits p47phox, p67phox, and p40phox, and the small GTPase Rac [8]. NOX2 is predominantly expressed in phagocytes, where it plays a crucial role in producing reactive oxygen species (ROS), a process commonly known as oxidative burst. Excessive ROS production can activate inflammasomes and mediate inflammatory responses [9,10]. Additionally, excessive ROS and/or impaired antioxidant responses can lead to oxidative stress, resulting in tissue injury and disrupted cell signaling [11]. Studies have shown that NOX2-deficient mice exhibit significantly reduced pulmonary fibrosis induced by bleomycin and decreased death of alveolar epithelial cells [12]. Moreover, inhibition of NOX2 reduced pulmonary fibrosis in a rat model of lung injury induced by bleomycin [13]. Mechanistically, NOX2 overexpression and ROS production can induce epithelial cell death, myofibroblast differentiation, and extracellular matrix (ECM) deposition.

The c-Jun N-terminal kinase (JNK) pathway is a subgroup of MAPK that is primarily activated by cytokines and environmental stressors. One of the main targets of the JNK signaling pathway is the activator protein-1 (AP-1) transcription factor, which is activated through the phosphorylation of c-Jun and related molecules [14,15]. Upon TGF-β1 binding to its receptor, phosphorylated receptors associate with TNF receptor-associated factor 6 (TRAF6) and transform growth factor-β-activated kinase 1 (TAK1), leading to the activation of mitogen-activated protein kinase kinase 4 (MKK4) and subsequent JNK phosphorylation, which results in the translocation of JNK into the nucleus. In the nucleus, JNK induces the phosphorylation of c-Jun and regulates target gene expression [16,17]. Some studies suggest that NOX2-mediated epithelial–mesenchymal transition (EMT) enhances JNK activity, promoting the proliferation, migration, and invasion of human epithelial cells in vitro [18,19]. Therefore, using JNK inhibitors to treat human epithelial cells may help block EMT while reversing the silica-induced proliferation, migration, and invasion of epithelial cells. This approach could be strategically important for alleviating tissue damage and treating fibrosis in silicosis patients.

In our study, we established a silicosis mouse model and performed single-cell sequencing on C57 mice at day 56. The sequencing results not only confirmed that silica exposure is a key factor in inducing pulmonary fibrosis but also showed increased expression of NOX2 and JNK in the silica-exposed group. These findings are consistent with previously published studies [9,10,18,19].

We found that the increase in ROS and oxidative stress produced by NOX2 in human lung epithelial BEAS-2B cells coincides with the increase in EMT. At the same time, overexpression of NOX2 can enhance the proliferation, wound closure, and migration ability of BEAS-2B cells. This indicates that SiO_2_-induced NOX2 expression can enhance JNK signal activity, ultimately leading to cell damage and promoting the EMT of lung epithelial cells. Therefore, reducing epithelial–mesenchymal transition by blocking silica-regulated JNK activation and inhibiting ROS produced by NOX2 is of great significance for the development of targeted therapies for silicosis patients.

This suggests that reducing epithelial–mesenchymal transition by blocking silica-regulated JNK activation and inhibiting NOX2-generated ROS could be of great significance in developing targeted therapies for silicosis patients.

## 2. Materials and Methods

### 2.1. Crystalline Silica

The crystalline silica (CAS 7631-86-9; particle size ranging from 0.5 to 10 μm, with 80% of the particles between 1 and 5 μm in diameter, and a purity of 99%) was sourced from Sigma-Aldrich Co., Ltd. (St. Louis, MO, USA). To eliminate endotoxins, the silica was subjected to heating at 200 °C for 2 h, followed by suspension in sterile phosphate-buffered saline (PBS). The silica suspension was treated with ultrasonic agitation for 10 min before application.

### 2.2. Establishment of a Mouse Model of Silicosis

Twenty-four healthy C57BL/6 mice (Male, 20–25 g) were procured from the experimental center of Ningxia Medical University. The mice were housed under controlled conditions, maintaining a stable temperature of 23 °C and 50% humidity, with a 12-h light/dark cycle. They were provided with food and water ad libitum. The mice were randomly assigned to two groups: (1) the control group mice received 50 μL of sterile saline via intratracheal administration, and (2) the silica group mice were intratracheally instilled with silica suspension (the dust had a particle size in the range of 0.5–10 μm, with 80% of the particles sized between 1–5 μm, and 50 mg/mL dissolved in saline). Lung tissues of mice were collected at 14 days, 28 days, and 56 days after the first injection of SiO_2_. The lungs were first rinsed with PBS, preserved in 4% formalin, dehydrated using a 30% sucrose solution, and then sliced and frozen for subsequent staining with hematoxylin-eosin. All animal procedures were fully compliant with the ARRIVE guidelines and received approval from the Institutional Animal Care and Use Committee at Ningxia Medical University’s Experimental Center (2023-G084).

### 2.3. Single-Cell RNA Library Construction and Sequencing

For the construction and sequencing of the single-cell RNA library, we utilized Cell Ranger software (10× Genomics, version 8.0.1) to align scRNA-Seq reads, aggregate unique molecular identifier (UMI) counts, identify individual cells, and normalize the transcriptome libraries. Following the manufacturer’s guidelines (10× Genomics), the Chromium system and the Single Cell 3 Reagent Kit (V1) were used to prepare barcoded scRNA-Seq libraries. Cells were clustered based on their expressed surface markers and categorized into distinct subgroups. Gene comparisons and subgroup naming were carried out, and each subset was assigned a name. Finally, Loupe Browser 7.0 was used for data visualization and analysis.

### 2.4. Cell Cultures and Treatments

BEAS-2B human bronchial epithelial cells (purchased from ATCC) were cultured in Dulbecco’s modified Eagle’s medium (DMEM)/Ham’s F12 medium (GIBCO, Paisley, UK) supplemented with 10% fetal bovine serum and 1% penicillin-streptomycin. The cells were maintained at 37 °C with 5% CO_2_. The experiment included a control group and a SiO_2_-treated group, where SiO_2_ particles (0.5–10 μm, 50 mg/mL in saline) were applied for 72 h. During cell culture, different reagents were added, including N-acetylcysteine (NAC, SIGMA, St. Louis, MO, USA) at concentrations of 1 μM and 5 μM, and the JNK inhibitor SP600125 (SELLECK, Houston, TX, USA) at 20 μM. Before SiO_2_ exposure, cells were pretreated with NAC for 1 h to reduce intracellular ROS levels or with SP600125 for 2 h to inhibit JNK expression.

### 2.5. Cell Scratch Assay

The scratch wound healing method was employed to evaluate cellular motility. Cells were plated in 6-well plates at a density of 1 × 10^6^ cells/mL and allowed to adhere for 12 h. After three washes with PBS, a scratch was made on the cell monolayer using a 1000 μL pipette tip. Detached cells were removed by rinsing three times with pre-warmed PBS. The wounded monolayer was then subjected to different experimental conditions and analyzed at various time points. Wound closure progression was monitored and recorded using a Leica microscope (Wetzlar, Germany). Cell migration ability was evaluated by calculating the migration index. All of our experiments were conducted in three separate biological experiments, and the data from these biological replicates were organized, statistically analyzed, and presented in Section 3.

### 2.6. Transwell Assay

Invasion assays were conducted using Transwell migration chambers (BD Biosciences, San Jose, CA, USA). The filters, with an 8 μm pore size, were coated with Matrigel (BD Biosciences, San Jose, CA, USA). The Matrigel was diluted to a 1:2 ratio using a serum-free medium and allowed to gel in an incubator for 30 min. A total of 2 × 10^4^ cells in 100 μL of culture medium were seeded in the upper chamber, while 700 μL of conditioned medium was added to the lower chamber. Cells were subjected to various experimental conditions at different time points. Calcium ions were removed from the culture medium by rinsing the cells twice with pre-cooled PBS. After fixation and two PBS washes, the cells were fixed with 4% paraformaldehyde for several minutes and then stained with 1% crystal violet for 20 min. All of our experiments were conducted in three separate biological experiments, and the data from these biological replicates were organized, statistically analyzed, and presented in Section 3.

### 2.7. Histological Staining

Tissue samples were evaluated using Hematoxylin and Eosin (HE) staining. Following dewaxing and hydration, sections were stained with hematoxylin for 15 min, rinsed with distilled water, and then stained with eosin for 7 min. After another rinse with distilled water, the sections were dehydrated and mounted. These stained sections were examined under a light microscope (BX-53; Olympus, Tokyo, Japan), and digital images were captured. Parameters such as trabecular bone volume (BV/TV), trabecular thickness (Tb.Th), trabecular number (Tb.N), and trabecular separation (Tb.Sp) were analyzed using Image Pro Plus 6.2 software (Media Cybernetics, Silver Spring, MD, USA). For quantitative assessment, three sections from each level (margin, center, and another margin) were selected to determine mean values. Comparisons were made between the control group (CON) and samples from corresponding levels, with average values of parallel samples used for analysis.

### 2.8. Immunofluorescence Staining

For immunofluorescence staining of BEAS-2B cells, sterile glass coverslips (VWR, Radnor, PA, USA) were placed in 24-well plates. Cells were seeded and exposed to SiO_2_ or saline controls under various experimental conditions at different time points. After washing three times with PBS, cells were fixed with 4% paraformaldehyde for 15 min and permeabilized with 0.1% Triton X-100 in PBS for 20 min at room temperature. Cells were then blocked with 5% bovine serum albumin (BSA; Sigma-Aldrich, St. Louis, MO, USA) for 1 h and washed with PBS. Subsequently, cells were incubated with α-SMA antibody (1:100, ab5694, Abcam, Cambridge, MA, USA) and Vimentin antibody (1:400, ab92547, Abcam, Cambridge, MA, USA) at 4 °C overnight. After rinsing with PBS three times for 5 min each, cells were incubated with a secondary antibody (FITC, 1:1000, Proteintech, Wuhan, China) for 1 h at room temperature. Nuclei were stained with 1.5 μM DAPI (blue). The primary and secondary antibodies used in this study are detailed in Table 1. All of our experiments were conducted in three separate biological experiments, and the data from these biological replicates were organized, statistically analyzed, and presented in Section 3.

### 2.9. Western Blot Analysis

Cells were washed three times with pre-chilled PBS and treated with 100 μL lysis buffer for 20 min. The lysate was centrifuged at 12,000 rpm for 15 min at 4 °C, and the supernatant was used for protein quantification via the BCA method. Thirty micrograms of protein from each group were separated by 10% SDS-polyacrylamide gel electrophoresis and transferred to a nitrocellulose membrane. The membrane was blocked with 5% skim milk and incubated with primary antibodies: E-Ca (1:1000, ab76055, Abcam, Cambridge, MA, USA), Vimentin (1:400, ab92547, Abcam, Cambridge, MA, USA), α-SMA (1:250, ab5694, Abcam, Cambridge, MA, USA), NOX2 (1:500, DF6520, Affinity Biosciences, Cincinnati, OH, USA), p-JNK (1:500, AF3318, Affinity Biosciences, Cincinnati, OH, USA), JNK (1:500, AF6318, Affinity Biosciences, Cincinnati, OH, USA), p-smad2/3 (1:1000, #8828, Cell Signaling Technology, Danvers, MA, USA), and smad2/3 (1:1000, #8685, Cell Signaling Technology, Danvers, MA, USA) at 4 °C overnight. After washing three times with Tris-Buffered Saline with Tween-20 (TBST), the membrane was incubated with secondary antibodies (Goat anti-rabbit IgG (H + L)/HRP or Goat anti-mouse IgG (H + L)/HRP) for 1 h at room temperature. Following additional washes with TBST, the membrane was treated with ECL (Thermo Fisher Scientific, Waltham, MA, USA) for visualization and quantification. The primary and secondary antibodies used in this study are detailed in Table 1. All of our experiments were conducted in three separate biological experiments, and the data from these biological replicates were organized, statistically analyzed, and presented in Section 3.

### 2.10. Wound Healing Assay

Cells were inoculated into 6-well plates at a density of 5 × 10^5^ cells/well and cultured to 90–100% confluence. A linear scratch (“wound”) was made on the monolayer of cells with a sterile 200 μL pipette tip. They were then rinsed twice with PBS to remove dislodged cells. Fresh medium (containing 2% FBS to minimize proliferative effects) was added and plates were incubated under standard conditions (37 °C, 5% CO_2_). Wound closure was monitored at 0 and 48 h using an inverted phase contrast microscope (10× objective). Images were acquired at the same locations using a motorized stage.

We used ImageJ software (version 1.53t, National Institutes of Health, Bethesda, MD, USA) for the quantitative analysis of wound width measurements. Data were the mean ± SD of three independent experiments (5 regions per replicate). Statistical significance was determined by two-way ANOVA and Tukey’s post hoc test (* *p* < 0.05, ** *p* < 0.01).

### 2.11. EdU and Ki67 Proliferation Assays

For EdU detection, we used the Invitrogen™ Click-iT™ EdU Imaging Kit (Alexa Fluor™ 647, Cat# C10340, Thermo Fisher Scientific, Waltham, MA, USA), following the manufacturer’s protocol. Cells were incubated with 10 μM EdU for 2 h, fixed, permeabilized, and stained using the Click-iT™ reaction cocktail. Hoechst 33342 was used for nuclear counterstaining, and images were captured using a fluorescence microscope.

For Ki67 detection, we performed standard immunofluorescence staining. Cells were fixed with 4% paraformaldehyde, permeabilized with 0.1% Triton X-100, and blocked with 5% BSA. Anti-Ki67 primary antibody was applied overnight at 4 °C, followed by incubation with fluorescent secondary antibodies. Nuclei were counterstained with DAPI and imaged under a fluorescence microscope.

### 2.12. Statistical Analysis

Data were derived from at least three independent experiments and expressed as mean ± standard deviation (SD). Differences between groups were analyzed using one-way analysis of variance (ANOVA), and comparisons between two groups were performed using the *t*-test. Statistical analysis was conducted using SPSS 21.0 software, with a *p*-value < 0.05 considered statistically significant.

## 3. Results

### 3.1. Generation of the Silicosis Mouse Model and Single-Cell Sequencing Classification of Mouse Lung

**Figure 1:**
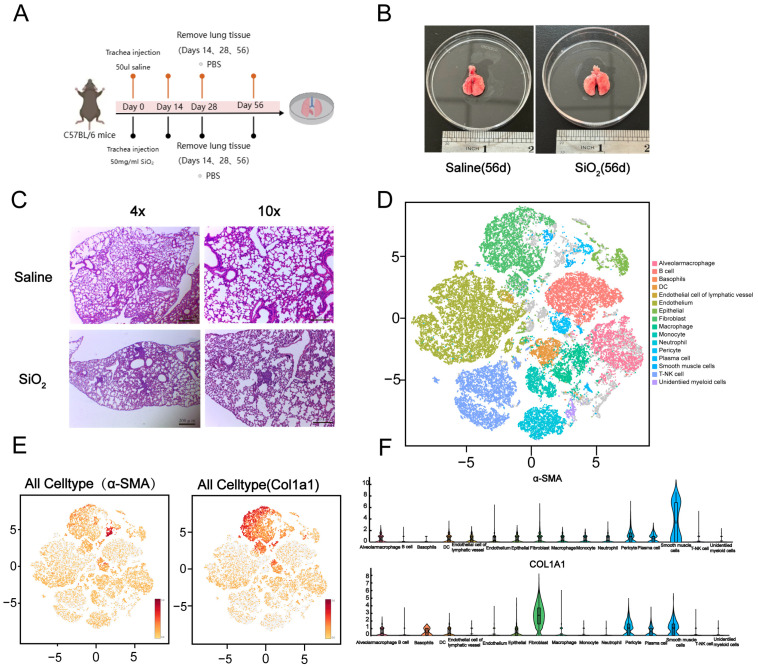
Development of a murine silicosis model and elevated expression of col1a1 and α-SMA in silicosis mouse lungs. (**A**) Six- to eight-week-old C57BL/6 mice were intratracheally administered 50 μL of crystalline silica dust (SiO_2_, 50 mg/mL in saline, with particle sizes ranging from 0.5–10 μm) or a saline control via the oropharyngeal route on days 1 and 7. Lungs from three male and three female mice per experimental group were collected at days 14, 28, and 56 following the initial silica dust administration. (**B**) Representative images captured at day 56 post-silica challenge demonstrated morphological changes in the lungs. (**C**) Histological examination using HE staining revealed an increased presence of silicotic nodules in lung sections of silica-treated mice at day 56. (**D**) Single-cell transcriptomic sequencing classified lung tissue cells into 16 distinct subtypes. (**E**,**F**) Pulmonary fibrosis model success was confirmed through single-cell sequencing, revealing elevated expression levels of col1a1 and α-SMA. These markers were used to assess the extent of silica-induced fibrosis.

Silicosis mouse models were established through intratracheal instillation via the oropharyngeal route using crystalline silica dust (50 mg/mL SiO_2_, 50 μL, with particle sizes ranging from 0.5–10 μm). Saline was used as the control and administered on days 1 and 7. The lungs of the mice were harvested at 14, 28, and 56 days post-initial silica dust administration (Figure 1A,B) [20,21]. HE staining was performed to investigate the pathophysiological progression in the silicosis mouse model. The results demonstrated that lung nodules were significantly more prominent in the silicosis-induced mice compared to the saline control group (Figure 1C). As recent studies suggest, various lung cell subtypes may play critical roles in driving pulmonary fibrosis and initiating pathological alterations in lung tissue for the experimental group [22]. Before detailed identification and characterization of lung cell subpopulations within healthy and fibrotic tissues, all the cells obtained from lung tissue homogenates were subjected to single-cell sequencing using the 10× Genomics Chromium platform. Sixteen lung tissue cell subtypes were identified based on cell surface markers (Figure 1D). Uniform Manifold Approximation and Projection (UMAP) analysis, along with violin plots, revealed elevated levels of α-SMA and collagen I expression—specific markers observed in silicosis models [23,24]. Single-cell sequencing analysis confirmed the heightened expression in the lungs of silicosis-induced mice (Figure 1E,F). Collectively, these findings strongly support the successful establishment of the silicosis mouse model.

### 3.2. Increased NOX2/JNK Expression and Epithelial-to-Mesenchymal Transition in the Small Airways of the Silicosis Model

**Figure 2:**
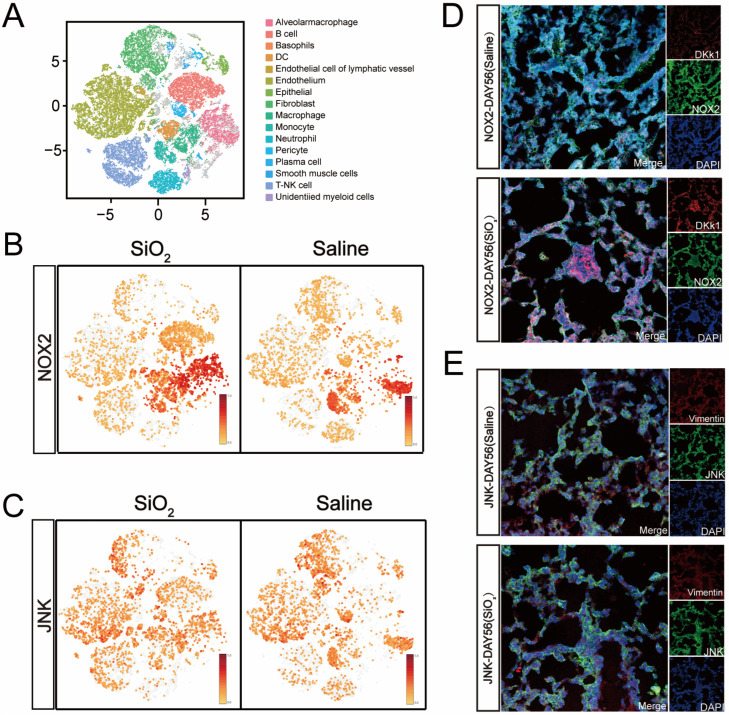
Depicts the elevated expression of NOX2 and JNK in the lungs of silicosis-afflicted mice induced by silica exposure at day 56. (**A**) The T-SNE plot, adapted from Figure 1D, showcases the expression patterns of the target markers NOX2 and JNK (**B**,**C**). These markers were analyzed across various lung cell subtypes in silica-induced silicosis mice at day 56 using single-cell sequencing, and their levels were compared to those of the saline-treated group (**B**,**C**). Immunofluorescence (IF) staining further verified the increased presence of NOX2 and JNK in the lung tissues at day 56 following initial exposure to silica dust (**D**,**E**). The scale bars in panels (**D**,**E**) measure 50 μm in both the first and second images.

Recent research using a ventilator-induced lung injury mouse model under hyperbaric oxygen or high tidal volume ventilation conditions has shown that NOX2 expression in lung epithelial and microvascular endothelial cells influences the activation of MAP kinase pathways, such as JNK and ERK1/2, as well as NF-kB signaling and associated transcriptional regulators such as c-Jun [25,26]. To explore the potential role of epithelial–mesenchymal transition (EMT) and pro-oxidase NOX2 in silicosis mice, the abundance of NOX2 and JNK proteins was examined through single-cell sequencing and immunofluorescence (IF) assays. Single-cell sequencing revealed a significant upregulation of NOX2 and JNK expression in the pulmonary tissues of mice with SiO_2_-induced silicosis at day 56 (Figure 2B,C). Similarly, IF assay results showed markedly increased expression of NOX2 and JNK following silica stimulation (Figure 2D,E). These findings strongly reaffirm that NOX2 plays a critical role in activating JNK signaling pathways.

### 3.3. NOX2-Derived ROS Is Essential for the Epithelial-to-Mesenchymal Transition (EMT) in Human Lung Epithelial Cells Induced by SiO_2_

**Figure 3:**
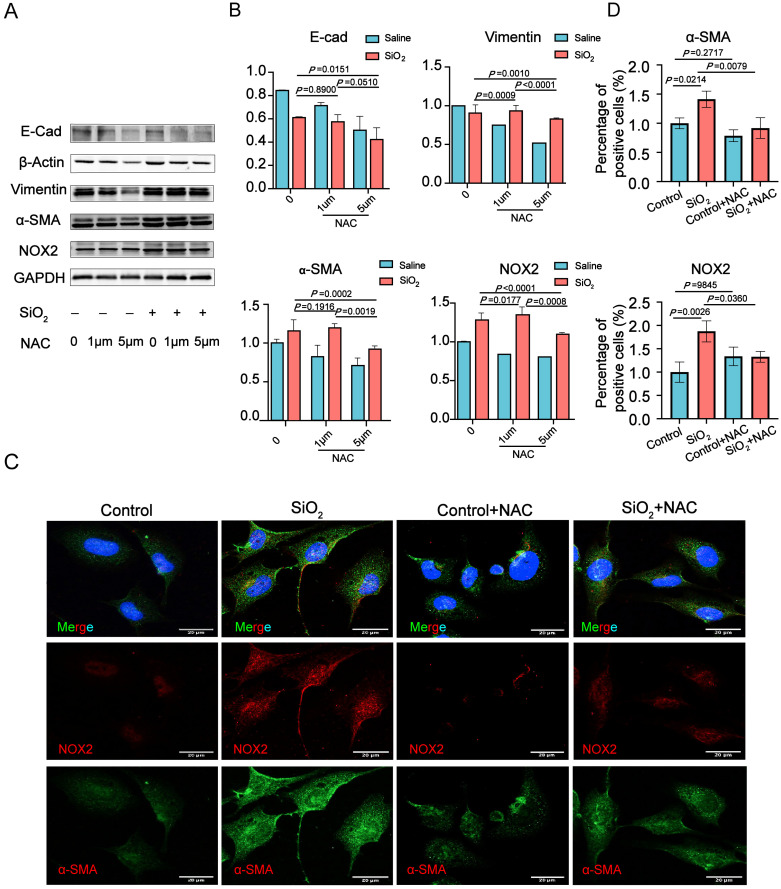
ROS scavenger N-Acetyl-L-cysteine (NAC) attenuates SiO_2_-induced NOX2 expression and EMT transition in human lung epithelial cells. BEAS-2B human bronchial epithelial cells were pre-incubated with varying concentrations of NAC (1–5 μM) for 1 h, followed by exposure to 100 μg/mL silica for 72 h.(**A**) Representative immunoblots revealed increased expression of NOX2 and EMT markers—E-cadherin (E-Cad), Vimentin, and α-SMA—in BEAS-2B cells treated with silica, as determined by immunoblotting assays. Notably, persistent NAC treatment (5 μM) significantly reduced silica-induced NOX2 and α-SMA expression in BEAS-2B cells compared to the silica-treated group. (**C**) Immunofluorescent analysis showed α-SMA (green) and NOX2 (red) in BEAS-2B cells exposed to saline or silica for 72 h, with or without NAC treatment (5 μM). Nuclei were labeled with DAPI (blue) to highlight the cellular structure in both untreated cells and cells exposed to 100 μg/mL SiO_2_ for 72 h. (**B**,**D**) Densitometric analysis was performed to quantify the relative protein levels in BEAS-2B cells treated with NAC and silica, as illustrated in panels (**A**,**C**). Data are presented as mean ± SD from three independent experiments, with comparisons made to both unstimulated controls and SiO_2_-treated controls. Representative staining images are displayed (*n* = 3), with a scale bar of 20 μm for all microscopy images.

As NADPH oxidases (NOXs) represent a key source of intracellular ROS production [27,28], and NOX2 plays a role in TGF-β1-induced epithelial-to-mesenchymal transition (EMT) [29,30], the expression levels of the NOX2 and EMT markers were analyzed using Western blot (WB) assays in BEAS-2B cells following SiO_2_ and NAC stimulation. A time-dependent upregulation of several mesenchymal markers was observed in BEAS-2B cells exposed to SiO_2_ for 24 to 96 h (Figure 3A,B). To evaluate the impact of altered ROS levels on BEAS-2B cells, human epithelial BEAS-2B cells were pretreated with the ROS scavenger NAC (1–5 μM) for 1 h prior to SiO_2_ exposure, followed by the detection of NOX2 and EMT marker proteins. The results revealed that NAC (5 μM) significantly reduced the levels of NOX2, E-cadherin, Vimentin, and α-SMA proteins (Figure 3A,B). Immunofluorescence findings further corroborated the WB results, confirming the SiO_2_-induced increases in α-SMA and NOX2 in BEAS-2B cells through immunofluorescence staining (Figure 3C). Additionally, NAC-induced decreases in intracellular ROS were associated with reduced NOX2 and α-SMA expression in BEAS-2B cells. Collectively, these findings suggest that reactive oxygen species (ROS) contribute to SiO_2_-induced NOX2 expression and regulate the epithelial–mesenchymal transition (EMT) process in BEAS-2B cells.

### 3.4. The Role of NOX2-Mediated JNK Signaling in Promoting EMT Transition in the Lung

**Figure 4:**
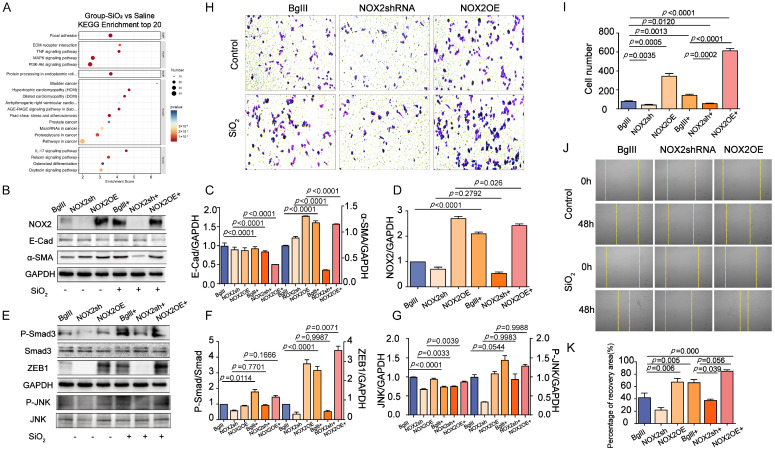
Effects of NOX2 on silica-induced EMT and JNK signaling in BEAS-2B Cells. BEAS-2B cells were infected with the specified adenoviral vectors at a multiplicity of infection (MOI) of 1000 for two hours, followed by exposure to silica and continued culture for 72 h. The expression levels of EMT marker molecules and phosphorylated JNK signaling were then evaluated. (**A**) The bubble chart highlights the involvement of the MAPK signaling pathway, which is closely associated with JNK activation. As a vital branch of the MAPK pathway, JNK plays a critical role in NOX2-mediated ROS production, driving silica-induced EMT and fibroblast activation in BEAS-2B cells. This underscores the importance of MAPK-JNK signaling in the pathological processes depicted. (**B**) Representative immunoblot images show the epithelial marker E-cadherin (E-cad), mesenchymal markers α-SMA, and NOX2 expression in BEAS-2B cells. Overexpression of NOX2 in BEAS-2B cells resulted in the upregulation of mesenchymal markers such as α-SMA and a reduction in the epithelial marker E-cad levels. In contrast, the shRNA-mediated knockdown of NOX2 produced the opposite effect. The experiments were performed in three biological replicates. (**C**,**D**) Quantitative analysis of the indicated protein expression in panel (**B**) was performed using densitometric analysis of immunoblots. (**E**) Representative immunoblots show phosphorylated JNK (Phos-JNK) signaling in BEAS-2B cells. Overexpression of NOX2 in BEAS-2B cells resulted in increased Phos-JNK expression, whereas shRNA-mediated NOX2 knockdown yielded the opposite effect. (**F**,**G**) The relative abundance of the indicated proteins in panel (**E**) was quantified via densitometric analysis of immunoblots across three independent experiments (*n* = 3). Representative invasion images of the above cells were assessed using the Transwell assay in BEAS-2B cells. Overexpression of NOX2 resulted in reduced cell migration ability and an increased cell count, while shRNA-mediated knockdown of NOX2 produced the opposite effect. Migration capacity was further evaluated using the cell scratch assay in BEAS-2B cells. (**H**,**I**) Transwell migration assay results for the control and silica-exposed (SiO_2_) groups treated with BGIII, NOX2 shRNA, and NOX2 overexpression (OE) are shown. The figure displays representative stained images and quantification of cell migration in Transwell assays (*n* = 3). (**J**,**K**) Quantification of wound gap closure in the wound healing assay is presented. The vertical yellow lines in the figure were used to mark the scratch wound healing process as an initial assessment of cell migration ability. The first row of the control and SiO_2_ groups represents the starting time (0 h), and the second row shows the wound changes at 48 h. In the NOX2 overexpression group, the wound appears significantly narrower at 48 h, indicating that NOX2 induction enhances the migration ability of BEAS-2B cells. The data are expressed as mean ± SD and are derived from three independent biological experiments conducted in triplicate (*n* = 3).

**Figure 5:**
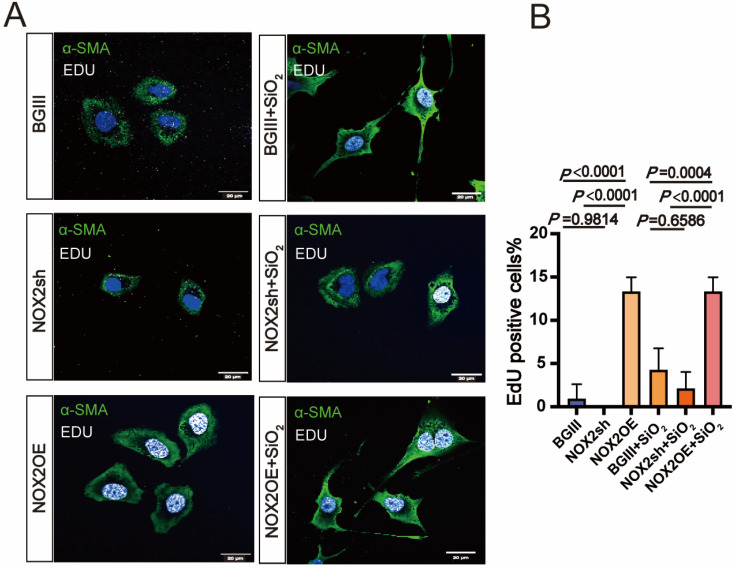
Inhibition of NOX2 leads to reduced proliferation of BEAS-2B cells. (**A**) Representative immunofluorescence images demonstrate α-SMA (green) expression and EdU (white) labeling in BEAS-2B cells treated with SiO_2_ for 72 h. Cells were infected with sh-NOX2 or OE-NOX2 constructs, and their performance was compared with those infected with the AdBglII control. Proliferative activity was assessed by quantifying the number of EdU-positive cells. The results indicate that overexpression of NOX2 enhances α-SMA expression and proliferative capacity in BEAS-2B cells, while shRNA-mediated suppression of NOX2 diminishes both α-SMA expression and proliferative capacity, regardless of SiO_2_ exposure. Expression levels were further quantified (**B**). The data are presented as mean ± SD across three independent biological experiments, each conducted in triplicate (*n* = 3).

To investigate whether NOX2 responds to the EMT transition in lung epithelial cells exposed to SiO_2_, its role in human lung epithelial BEAS-2B cells was modulated via adenoviral vectors. Adenoviral vectors encoding NOX2 (Ad.NOX2) were utilized to achieve overexpression, while short-hairpin RNA (shRNA) targeting NOX2 (Ad.shRNA) was used for knockdown. As expected, BEAS-2B cells infected with Ad.NOX2 displayed elevated NOX2 protein levels, whereas those infected with Ad.shRNA showed a significant reduction in NOX2 expression. These results confirm that the adenoviral vectors effectively mediated NOX2 overexpression and knockdown in BEAS-2B cells (Figure 4). The Ad.NOX2-mediated overexpression of NOX2 activated EMT, as evidenced by decreased levels of the epithelial marker E-cadherin and increased levels of the profibrogenic factor alpha-smooth muscle actin (a-SMA) in human epithelial cells (Figure 4B–D). Additionally, cells infected with Ad.NOX2 exhibited higher levels of SMAD family member 3 (Smad3), phosphorylated SMAD family member 3 (P-Smad3), and Zinc finger E-box binding homeobox 1 (ZEB1) (Figure 4E,F). KEGG enrichment analysis revealed notable enrichment of the MAPK signaling pathway in the SiO_2_-treated group, with JNK identified as a key component. As a pivotal branch of the MAPK pathway, JNK played a central role in NOX2-mediated EMT and fibroblast activation. Ad.NOX2-infected cells showed increased JNK signaling activity, characterized by elevated phosphorylated JNK (Phos-JNK) expression (Figure 4A,E,G). Consistently, immunostaining demonstrated elevated a-SMA expression in BEAS-2B cells infected with Ad.NOX2, independent of SiO_2_ exposure (Figure 5A). Conversely, the protein levels in cells infected with Ad.shNOX2 exhibited an inverse pattern compared to those overexpressing NOX2 (Figure 5A). Overexpression of NOX2 enhanced the proliferative, wound closure, and migration capacities of BEAS-2B cells infected with Ad.NOX2, as evidenced by immunostaining from the scratch assay and Transwell assay, regardless of SiO_2_ exposure. Knockdown of NOX2 reduced these capacities in BEAS-2B cells (Figure 4H,I). The scratch wound healing assay was employed to assess the rate of wound closure and cell migration. This effect became more pronounced at 48 h, with the percentage of the scratch area at 48 h serving as a more precise measure of cell migration in comparison to the initial time point (Figure 4J,K). Collectively, these findings indicate that SiO_2_-induced NOX2 expression enhances JNK signaling activity, ultimately causing cell injury and promoting EMT in lung epithelial cells.

### 3.5. Inhibition of JNK/C-Jun Signaling by SP600125 Effectively Reversed the Proliferation, Migration, and Invasion Capacities Induced by SiO_2_ in Human Epithelial Cells

**Figure 6:**
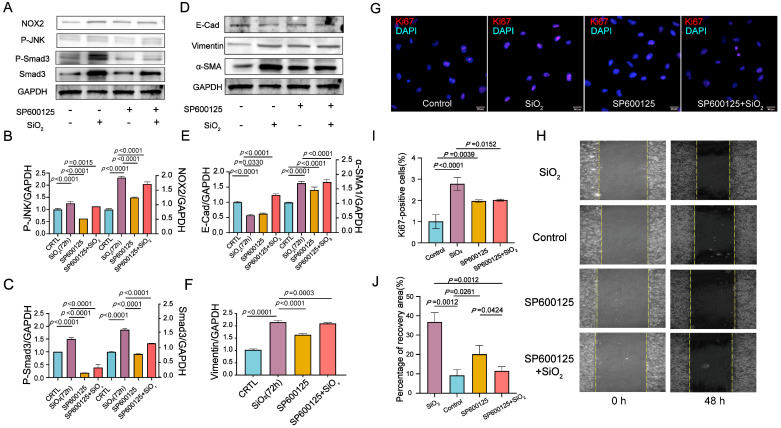
NOX2 activates EMT transition via JNK/c-Jun signaling to promote epithelial cell proliferation, migration, and invasion. JNK inhibitor SP600125 reverses SiO_2_-induced proliferation, migration, and invasive capacity in human epithelial cells. BEAS-2B cells were pretreated with 20 μM SP600125 for 2 h, followed by exposure to SiO_2_ and subsequent culture for 72 h. Epithelial-to-mesenchymal transition (EMT) and cell proliferation were analyzed using immunoblotting and immunofluorescence staining. (**A**) Representative immunoblots demonstrate activation of the JNK signaling pathway in SiO_2_-exposed BEAS-2B cells, while SP600125 markedly inhibits JNK signaling activation. This effect is evidenced by decreased expression of key pathway components, including p-JNK and p-c-Jun. Additionally, the TGF-β/Smad signaling component p-Smad3 shows reduced levels in BEAS-2B cells pretreated with SP600125 compared to those treated solely with SiO_2_. (**B**,**C**) Quantitative densitometric analysis of the immunoblot results in (**A**) indicates the relative abundance of specified proteins. (**D**) BEAS-2B cells pretreated with SP600125 displayed a reduction in the abundance of mesenchymal markers, such as Vimentin and SMA, alongside increased expression of the epithelial marker E-cadherin (E-Cad), as demonstrated by Western blot analysis. (**E**,**F**) Quantitative densitometric analysis of protein expression in (**D**) further corroborates these findings. (**G**,**I**) Immunofluorescence staining for the proliferation marker Ki67 (red) illustrates diminished proliferative capacity in SP600125-pretreated BEAS-2B cells compared to SiO_2_-treated cells. Quantification of Ki67-positive cells supports a significant reduction in proliferation. (**H**) The migratory capacity of BEAS-2B cells was assessed via a cell scratch assay. Representative images demonstrate reduced cell migration in SP600125-pretreated cells compared to SiO_2_ controls. (**J**) Quantification of the wound closure in the cell scratch assay confirms attenuated migratory behavior in the SP600125-treated group. Data are presented as the mean ± SD of three independent biological experiments, each performed in triplicate (*n* = 3).

To investigate the effects of JNK signaling on SiO_2_-induced NOX2 expression and EMT-mediated cell proliferation, migration, and invasion in human epithelial cells, the activated Phos-JNK and NOX2 protein expression were suppressed, and the SiO_2_-mediated EMT transition was reversed. This reversal was evidenced by a decreased abundance of α-SMA and P-Smad3, alongside an increased level of E-Cad in BEAS-2B cells pretreated with SP600125 (Figure 6A–F). Scratch wound healing assays were performed to evaluate both the wound closure rate and the number of migrating cells. SiO_2_ treatment promoted wound closure in human epithelial cells; however, pretreatment with SP600125 significantly delayed SiO_2_-induced cell migration. This delay became more pronounced at 48 h. The percentage of the scratched area at 48 h compared to that at 0 h was calculated to quantify the rate of cell migration during the wound healing assay (Figure 6I,J). Consistently, the proliferation capacity was assessed through immunostaining, showing an increased abundance of Ki67-positive cells in SiO_2_-treated epithelial cells. In contrast, there were fewer Ki67-positive cells in cultures pretreated with SP600125 compared to the SiO_2_-treated cells (Figure 6G,H). These findings suggest that inhibiting the JNK/C-Jun signaling pathway with SP600125 effectively reverses the proliferation, migration, and invasion capacities induced by SiO_2_ in human epithelial cells.

## 4. Discussion

This study examines NOX2-mediated ROS production in SiO_2_-induced epithelial–mesenchymal transition (EMT) and investigates the interplay between NOX2 and the JNK signaling pathway following SiO_2_ exposure. The findings indicate that SiO_2_-induced EMT can be markedly mitigated by suppressing NOX2-derived ROS in human epithelial cells, which subsequently inhibits the activation of the JNK signaling pathway. Moreover, employing NOX2 siRNA and a JNK inhibitor to reduce the expression of NOX2 and JNK in human epithelial cells reveals a positive feedback mechanism between these pathways during SiO_2_-induced EMT.

EMT induction is widely acknowledged as a pivotal factor in the progression of fibrotic diseases, such as silicosis. This process entails the transformation of epithelial cells into a mesenchymal-like phenotype, which significantly contributes to fibrosis and tissue remodeling [31]. In liver fibrosis, the activation of the TGF-β signaling pathway triggers the conversion of liver epithelial cells into mesenchymal cells, thereby aggravating fibrous tissue deposition and accelerating liver fibrosis progression [32]. Similar phenomena are observed in renal fibrosis, where renal tubular epithelial cells transition into fibroblast-like cells under the stimulation of inflammatory factors (e.g., TGF-β, IL-1β). This transition leads to the production of abundant extracellular matrix components (such as collagen), resulting in abnormal deposition within the renal interstitium, the disruption of renal tubular structures, and ultimately, renal fibrosis [33]. By using some natural drugs such as *Dendrobium officinale* (DOE) on myocardial fibrosis in mice, DOE can suppress fibrosis by inhibiting EMT and EMT-related signaling molecules (such as TGF-β1, p-JNK, Twist, Snail1, Vimentin, etc.) [34]. These findings collectively underscore that EMT is a key pathological driver in the development and progression of fibrosis. In our study, we established a silicosis mouse model and assessed relevant markers to further investigate the EMT process. Western blotting and single-cell sequencing were used to detect epithelial and mesenchymal cell markers, including E-cadherin (E-Cad), α-SMA, Vimentin, and the transcription factor ZEB1. The results revealed a reduction in the epithelial marker E-Cad, while the expression levels of the mesenchymal markers α-SMA, Vimentin, and ZEB1 were notably elevated in the lung tissue of silicosis mice. These findings indicate an amplified EMT process in the lung tissue following exposure to SiO_2_.

Repeated oxidative stress plays a pivotal role in lung injury. Reactive oxygen species (ROS) not only drive the epithelial-to-mesenchymal transition (EMT) of lung epithelial cells, leading to fibrosis, but also disrupt alveolar cell homeostasis. The overproduction of ROS, mediated by NOX enzymes and triggered by excessive stimulation from pro-inflammatory cytokines or environmental factors such as silica dust, is a major contributor to oxidative stress in the lungs. Among the various NOX isoforms, NOX2 emerges as a critical enzyme integral to epithelial cell transition, fibroblast proliferation, and collagen deposition [35,36]. Experimental studies demonstrate that NOX2 overexpression significantly elevates ROS production, thereby exacerbating oxidative stress, especially in diabetic patients. Research has revealed that proanthocyanidin-loaded PMS nanoparticles downregulate NOX2 expression, curbing ROS overproduction, reducing oxidative stress, and promoting osteoblast differentiation and ossification [37]. Collectively, these findings underscore the role of NOX2 in amplifying oxidative stress through ROS generation, suggesting that NOX2 could be a promising therapeutic target for treating pulmonary fibrosis, including silicosis [38,39]. In lung macrophages and neutrophils, NOX2-driven ROS generation plays a dual role: aiding in pathogen defense while contributing to inflammation, oxidative stress, and tissue damage, particularly in chronic obstructive pulmonary disease (COPD) and idiopathic pulmonary fibrosis (IPF) [9,40,41,42,43,44]. Some studies have focused on intrauterine growth restriction by C57 mice to inhibit NOX2, and NOX2 affects VEGF-A expression and angiogenesis through ROS [45]. Similarly, the NOX2-specific inhibitor GSK2795039 has been used to investigate its role in atherosclerosis, revealing that ROS generated by NOX2 affects macrophage phagocytic function and the stability of vulnerable plaques [46]. These findings collectively indicate that inhibiting NOX2 could mitigate ROS production, reduce myofibroblast activation, limit extracellular matrix component accumulation, and slow the progression of pulmonary fibrosis [43,47]. Such studies suggest that targeting oxidative stress via NOX2 inhibition has potential as an effective strategy for managing IPF and other pulmonary diseases, including silicosis. Our investigation revealed elevated NOX2 expression and increased ROS production in lung epithelial cells exposed to SiO_2_. Treatment with the antioxidant NAC notably curtailed ROS production, while shRNA-mediated NOX2 knockdown significantly reduced SiO_2_-induced fibrogenic markers (α-SMA, vimentin) in lung epithelial cells. These findings highlight NOX2 and ROS as key contributors to the development and pathogenesis of disease. Regulating NOX2 expression could effectively curb ROS overproduction, thereby mitigating the detrimental effects of oxidative stress. The JNK signaling pathway is integral to numerous cellular functions and is pivotal in regulating various cellular processes, including inflammation, apoptosis, and fibrosis. Recent studies have elucidated the multifaceted functions of JNK signaling in disease development and progression. Dehydrocostus lactone (DHL), a bioactive substance derived from the rhizome of *Saussurea lappa*, is recognized for its anti-inflammatory, immunomodulatory, and anti-fibrotic effects, particularly in managing pulmonary fibrosis. Studies indicate that DHL attenuates pulmonary fibrosis in a bleomycin-induced mouse model by reducing TLR4 expression and inhibiting JNK and p38 MAPK signaling pathways [48]. These findings align with prior research demonstrating that elevated JNK phosphorylation is a key driver of pulmonary fibrosis by promoting EMT differentiation in human lungs [49]. A growing body of literature underscores the interplay between the JNK signaling pathway and ROS regulation in cancer, neurodegenerative diseases, cardiovascular diseases, diabetes, and metabolic disorders [50,51,52,53,54]. Research has shown that ROS produced by NOX2 activates P2X7 receptor-mediated JNK signaling in reproductive-related diseases, contributing to granulosa cell inflammation and apoptosis in polycystic ovary syndrome (PCOS) [55]. Furthermore, ROS-induced activation of p38 and JNK has been implicated in apoptosis induction through intrinsic pathways in colorectal cancer cells, offering potential applications in cancer therapy, particularly for chemotherapy-resistant cancers such as oxaliplatin-resistant cells [56]. Emmy Hainida Khairul Ikram and colleagues have examined the role of natural antioxidants in modulating the JNK signaling pathway by regulating ROS levels. Notably, activation of NF-κB was found to inhibit ROS-induced apoptosis by suppressing JNK signaling activity [57]. However, despite these advances, research on the relationship between the JNK pathway and ROS in silicosis remains limited. The mechanisms by which NOX2 regulates JNK signaling, induces EMT in silicosis, and contributes to its pathogenesis remain unclear and warrant further investigation.

This study revealed that NOX2 is significantly upregulated following SiO_2_ exposure, facilitating the EMT transformation of lung epithelial cells and associated functional changes through the JNK signaling pathway. Our data demonstrate that the shRNA-mediated knockdown of NOX2 markedly reduces ROS production, thereby diminishing SiO_2_-induced activation of the JNK signaling pathway in BEAS-2B cells. This aligns with the established understanding that JNK serves as a downstream effector in ROS-mediated signaling pathways [58]. ROS-induced cell death and activation of the JNK pathway were triggered by a sulfonamide derivative. Moreover, we identified that JNK promotes the expression of transcription factors such as ZEB1, which play pivotal roles as regulators of EMT. NOX2 overexpression enhances SiO_2_-induced EMT in lung epithelial cells by activating the JNK signaling pathway while concurrently boosting cell proliferation, migration, and invasion. The application of SP600125, an inhibitor of the JNK/C-Jun signaling pathway, significantly counteracted SiO_2_-induced NOX2 elevation and suppressed cell proliferation and migration in BEAS-2B cells. This was evidenced by the reversal of molecular changes, including the downregulation of E-cadherin and the upregulation of α-SMA, P-Smad3, and ZEB1—changes closely tied to JNK signaling activation. Through the use of SP600125, we successfully mitigated the SiO_2_-induced upregulation of NOX2, EMT transformation, and enhanced cell proliferation and migration. These findings underscore the critical role of the JNK signaling pathway in NOX2-mediated EMT transformation of lung epithelial cells and suggest novel therapeutic targets for the treatment of silicosis. Notably, this mechanism parallels research on liver fibrosis, where the inhibition of ROS levels and NOX2 expression in the liver was shown to reduce collagen production and suppress the activation of ERK and JNK signaling pathways, thereby halting the progression of liver fibrosis [59].

Consequently, targeted blockage of the JNK signaling pathway may represent an effective strategy to mitigate oxidative stress and inhibit the advancement of liver fibrosis. This study underscores the pivotal role of NOX2-derived ROS in driving SiO_2_-induced EMT via activation of the JNK pathway. These findings may offer valuable insights and strategies for identifying key drivers and mechanisms underlying lung fibrosis in silicosis, shedding light on its pathogenesis and progression. Furthermore, they pave the way for exploring molecular targets within the NOX2-JNK signaling pathway to combat fibrotic diseases by inhibiting EMT. Nonetheless, the therapeutic potential and precise mechanisms of NOX2 and JNK inhibitors remain to be fully investigated, particularly regarding their safety and efficacy, which must be rigorously evaluated before clinical application.

## 5. Conclusions

In conclusion, this study highlights a significant crosstalk between JNK signaling and NOX2 in the context of silica dust-induced lung injury, particularly in the pathogenesis of silicosis. The positive feedback loop between JNK and NOX2 not only promotes ROS production and EMT but also exacerbates lung epithelial cell injury. These findings suggest that targeting the interaction between JNK and NOX2 could be a potential therapeutic strategy to mitigate the progression of silicosis and other related pulmonary fibrotic diseases.

## Figures and Tables

**Table 1:** Primary and secondary antibodies used in this project.

Antibody	Cat #	Dilution Ratio			Company
		WB	IHC	IF	
α-SMA	Ab5694	1:2000	-	1:200	Abcam, Cambridge, MA, USA
NOX2	DF6520	1:1000	-	1:200	Affinity, Changzhou, China
JNK	AF6318	1:1000	-	-	Affinity, Cincinnati, OH, USA
P-JNK	AF3318	1:1000	-	-	Affinity, Cincinnati, OH, USA
P-C-Jun	AF3095	1:1000	-	-	Affinity, Cincinnati, OH, USA
E-Cad	Ab76055	1:1000	-	-	Abcam, Cambridge, MA, USA
E-Cad	AF0131	1:2000	-	-	Affinity, Changzhou, China
Vimentin	Ab92547	1:1000	-	1:200	Abcam, Cambridge, MA, USA
Smad2/3	#8685	1:1000	-	-	Cell Signaling Technology (CST), Danvers, MA, USA
P-Smad2/3	#8828	1:1000	-	-	Cell Signaling Technology (CST), Danvers, MA, USA
Zeb1	DF7414	1:1000	-	-	Affinity, Cincinnati, OH, USA
anti-GAPDH	AF7021	1:2000	-	-	Affinity, Changzhou, China
Goat anti-rabbit IgG (H + L)/HRP antibody	S0001	1:1000	-	-	Affinity, Cincinnati, OH, USA
Goat anti-mouse IgG (H + L)/HRP antibody	S0002	1:1000	-	-	Abcam, Cambridge, MA, USA
Anti-collagen 1 antibody	Ab34710	1:5000	-	-	Abcam, Cambridge, MA, USA
488-conjugated donkey anti-mouse	711-545-150	-	-	1:200	Jackson ImmunoResearch, West Grove, PA, USA
647-conjugated donkey anti-rabbit	711-605-152	-	-	1:200	Jackson ImmunoResearch, West Grove, PA, USA

## Data Availability

Original research data described in the article are available upon request.
